# MLAD: A Multi-Task Learning Framework for Anomaly Detection

**DOI:** 10.3390/s25134115

**Published:** 2025-07-01

**Authors:** Kunqi Li, Zhiqin Tang, Shuming Liang, Zhidong Li, Bin Liang

**Affiliations:** Faculty of Engineering and IT, University of Technology Sydney, 15 Broadway, Sydney, NSW 2007, Australia; kunqi.li@student.uts.edu.au (K.L.); zhiqin.tang-1@student.uts.edu.au (Z.T.); shuming.liang@uts.edu.au (S.L.); zhidong.li@uts.edu.au (Z.L.)

**Keywords:** anomaly detection, multi-task learning, multivariate time series, sensor clustering, graph neural networks

## Abstract

Anomaly detection in multivariate time series is a critical task across a range of real-world domains, such as industrial automation and the internet of things. These environments are generally monitored by various types of sensors that produce complex, high-dimensional time-series data with intricate cross-sensor dependencies. While existing methods often utilize sequence modeling or graph neural networks to capture global sensor relationships, they typically treat all sensors uniformly—potentially overlooking the benefit of grouping sensors with similar temporal patterns. To this end, we propose a novel framework called Multi-task Learning Anomaly Detection (MLAD), which leverages clustering techniques to group sensors based on their temporal characteristics, and employs a multi-task learning paradigm to jointly capture both shared patterns across all sensors and specialized patterns within each cluster. MLAD consists of four key modules: (1) sensor clustering based on sensors’ time series, (2) representation learning with a cluster-constrained graph neural network, (3) multi-task forecasting with shared and cluster-specific learning layers, and (4) anomaly scoring. Extensive experiments on three public datasets demonstrate that MLAD achieves superior detection performance over state-of-the-art baselines. Ablation studies further validate the effectiveness of the modules of our MLAD. This study highlights the value of incorporating sensor heterogeneity into model design, which contributes to more accurate and robust anomaly detection in sensor-based monitoring systems.

## 1. Introduction

From the internet of things to industrial automation, multivariate time-series data power decision-making by capturing dynamic system behavior [1,2]. Anomaly detection is a fundamental task in these data-driven systems, aiming to identify patterns or observations that deviate from expected behavior. It is critical across various domains, including detecting power grid failures [2], ensuring autonomous vehicle safety [3], identifying anomalies in railway infrastructure [4], and monitoring water treatment systems [5].

**Figure 1:**
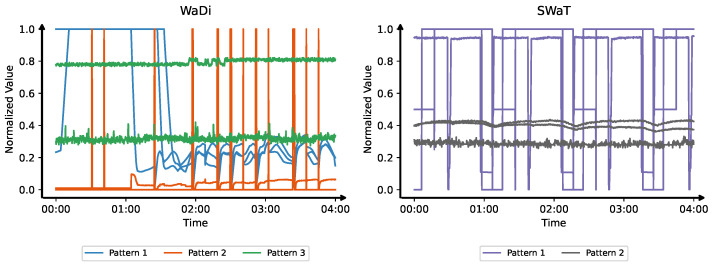
Temporal pattern diversity across sensor subsets and consistency within each subset in two datasets. (**Left**): Water Distribution (WaDi) [7]; (**Right**): Secure Water Treatment (SWaT) [8]. In WaDi, Pattern 1 (blue) includes sensors with smooth, gradual trends; Pattern 2 (orange) shows binary transitions; Pattern 3 (green) exhibits steady-state signals. In SWaT, Pattern 1 (purple) features on/off toggling, while Pattern 2 (gray) contains stable analog measurements.

In real-world industrial deployments, anomaly detection systems typically rely on large-scale, heterogeneous sensor networks to continuously monitor various subsystems and operational parameters [6]. These sensors exhibit a wide diversity of temporal behaviors due to differences in their physical characteristics and functional roles. However, despite this diversity, sensors within the same functional class often produce similar temporal patterns. As shown in Figure 1, sensors in Water Distribution (WaDi) [7] and Secure Water Treatment (SWaT) [8] datasets display recurring class-specific signatures, such as gradually rising and falling values, sharp and frequent switches between high and low states, and nearly constant signals with minimal variation. These characteristic patterns among functionally or behaviorally similar sensors suggest the potential benefit of modeling sensor subsets based on temporal similarity.

Despite recent advances for multivariate time-series anomaly detection [1,9], most existing methods adopt a uniform modeling strategy that treats all sensors equally. For example, recent Graph Neural Network (GNN)-based models [10,11] achieve state-of-the-art performance. These approaches typically construct a global adjacency matrix to capture correlations among all sensors and apply spatial–temporal modeling over the entire graph. However, this design overlooks the fact that many sensors consistently exhibit similar temporal behaviors within a specific subset of sensors. As a result, the models tend to learn only global patterns shared across the entire sensor system, while neglecting the specific temporal patterns embedded within a particular family of sensors. This can lead to reduced sensitivity to localized anomalies that are only apparent within certain classes of sensors.

To address these limitations, we propose a **M**ultitask **L**earning-based **A**nomally **D**etection framework, termed **MLAD**. We build MLAD with four key components: (1) sensors clustering—we perform unsupervised clustering to classify sensors into subsets based on similarities in their time series; (2) time-series representation learning with a cluster-constrained GNN—a graph is constructed in which edges are restricted to connections within each sensor cluster, which allows the GNN to focus on learning relationships specific to individual clusters, rather than modeling the entire sensor network uniformly; (3) multi-task learning—the learned representations from the GNN are then passed into a multi-task learning module that comprises both shared layers for capturing global patterns across all sensors, and task-specific (i.e., cluster-specific) layers that learn local temporal dynamics unique to each sensor cluster; and (4) anomaly detection—anomalies are finally detected by a Principal Component Analysis (PCA)-based reconstruction errors over forecasting errors. Extensive experiments on three real-world datasets demonstrate that MLAD consistently outperforms strong baselines. Ablation studies confirm the effectiveness of both the cluster-constrained graph neural networks and multi-task forecasting module.

Our main contributions are summarized as follows:

- We employ an unsupervised clustering strategy to group sensors into clusters and introduce a cluster-constrained GNN that enables the model to focus on sensor relationships within each cluster.
- We introduce a multi-task forecasting architecture to multivariate time-series anomaly detection that jointly learns global behaviors shared across all sensors and specialized patterns unique to each cluster of sensors, which finally benefits the performance of the downstream anomaly detection task.
- Extensive experiments on three public datasets show that MLAD outperforms state-of-the-art baselines, and ablation studies confirm the contribution of each module to its strong detection performance.

The remainder of this paper is organized as follows. Section 2 reviews related works on multivariate time-series anomaly detection. Section 3 formally defines the problem of multivariate time-series anomaly detection. Section 4 details our proposed framework, including sensor clustering, cluster-constrained GNN, multi-task learning, and anomaly detection. Section 5 presents experimental settings and results, followed by discussions. Finally, Section 6 concludes the paper and outlines future research directions.

## 2. Related Work

### 2.1. Time-Series Anomaly Detection

Anomaly detection in multivariate time series has been a longstanding research challenge in multiple domains such as industrial automation and internet of things. Traditional statistical methods, including autoregressive integrated moving average [12], PCA [13], and support vector machines [14], have been widely applied to this field. Despite their interpretability and low computational cost, these traditional models have several key limitations [15]. First, they struggle with handling non-linear and high-dimensional dependencies. Second, statistical models generally rely on strong assumptions, such as stationarity and independence, which do not hold in many real-world situations.

With the limitations of traditional statistical methods, deep learning has emerged as a powerful alternative for analyzing and detecting anomalies in multivariate time-series data. Specifically, deep learning models offer three key advantages over classical approaches: automatic feature extraction, non-linearity modeling, and adaptability to large-scale data [9]. Furthermore, deep learning models do not rely on strong stationarity assumptions, making them more suitable for real-world, dynamic datasets where time-series patterns evolve over time.

Among the widely used deep learning architectures for time-series anomaly detection, Recurrent Neural Networks (RNNs), Convolutional Neural Networks (CNNs), and Variational Autoencoders (VAEs) each offer distinct modeling capabilities. RNNs, particularly long short-term memory networks, are well-suited for capturing long-range temporal dependencies in sequential data [16]. CNNs are designed to extract local and hierarchical patterns by applying convolutional filters over the input. When applied to time series, CNNs can effectively capture short-term dependencies and spatial correlations between variables, making them a powerful alternative or complement to RNNs in capturing complex dynamics [17]. Meanwhile, VAEs offer a generative approach that learns a probabilistic latent representation of the input data and detecting anomalies by reconstructing normal patterns and quantifying deviations through reconstruction errors [18].

Despite notable success, deep learning approaches still face key challenges. Traditional RNN-, CNN-, and VAE-based methods do not explicitly model relationships between different time-series variables, instead encoding them into global hidden states [11]. This lack of modeling relationship between variables can lead to suboptimal performance, particularly in complex, high-dimensional multivariate time-series settings. As a result, GNNs have recently gained attention as a promising approach for anomaly detection by explicitly representing dependencies between time-series variables.

### 2.2. Graph Neural Networks

Graph Neural Networks offer a solution to explicitly modeling dependencies among time-series variables as graph structures [19]. The key idea behind GNNs is the message-passing mechanism, where each node (representing a time-series variable or sensor) iteratively updates its representation by aggregating information from its neighboring nodes. This enables the model to learn meaningful spatial relationships between different variables while simultaneously capturing temporal dynamics.

As a result, GNN-based anomaly detection methods have shown promising results in detecting complex anomalies that involve interactions between multiple variables. Deng and Hooi [10] introduced a structure-learning-based GNN with attention mechanisms to explicitly model inter-sensor relationships, enhancing both detection accuracy and explainability. Ning et al. [20] expanded on this by proposing MST-GNN, which integrates multi-scale temporal features with GNNs to capture latent correlations, significantly improving anomaly detection performance. Zheng et al. [11] further advanced the field with CST-GL, a correlation-aware spatial–temporal graph learning model that leverages graph convolution to encode rich spatial information from complex pairwise dependencies between variables.

Despite their success, GNN-based anomaly detection models face a critical challenge: balancing the need to capture global dependencies across the sensor network while preserving the distinct, localized behaviors of functionally similar sensors. Most existing approaches construct a global adjacency matrix across all sensors, which may obscure meaningful dynamics specific to individual sensor groups. This design often favors general patterns at the expense of sensors’ cluster-specific characteristics, limiting the model’s ability to detect anomalies.

To address this limitation, we innovatively proposed a promising direction is to incorporate sensor cluster information through unsupervised clustering and cluster-constrained graph design. By first identifying clusters among sensors based on temporal similarity, models can construct structured priors that more accurately reflect subsystem boundaries. These clusters can then guide both the construction of the sensor graph and the design of forecasting modules, enabling more fine-grained modeling.

### 2.3. Multi-Task Learning

Multi-Task Learning (MTL) is an increasingly prominent paradigm in machine learning wherein a single model is trained to simultaneously solve multiple related tasks [21]. By leveraging shared representations across tasks, MTL promotes inductive transfer, which can lead to improved generalization, faster convergence during training, and enhanced robustness, particularly in data-scarce scenarios [22]. This shared structure enables the model to learn commonalities and distinctions across tasks, often outperforming models trained in isolation on each task.

MTL has demonstrated broad applicability across various domains. In natural language processing, it has been employed for joint tasks such as part-of-speech tagging and named entity recognition [23]. In computer vision, MTL enables models to perform complex tasks like egocentric vision in data-scarce scenarios [24]. In recommender systems, MTL has been employed to jointly model interactions from multiple domains, such as user–item and user–social relationships, thereby improving performance in cold-start settings [25]. A common architectural approach in MTL involves a shared feature extraction module—often based on CNNs or transformer encoders—followed by task-specific output heads that tailor predictions to individual objectives [23]. This design balances parameter sharing with task-specific specialization, capturing both general and unique aspects of each task.

In this work, we build upon the multi-task learning paradigm by proposing a novel time-series forecasting module, specifically designed for heterogeneous, multivariate time-series data. Our approach define forecasting tasks based on sensor clusters—each representing a coherent temporal pattern. The forecasting module incorporates cluster-specific layers to adapt its output processing to the nuances of each sensor cluster, while retaining shared layers across all sensors to encourage joint optimization. This design can foster localized specialization of each sensor clusters without forfeiting the benefits of shared representations captured by the cluster-constrained CNN model.

## 3. Problem Statement

The problem addressed in this paper is multivariate time-series anomaly detection, where each time step comprises simultaneous observations of *N* correlated variables. Such data commonly arise in sensor networks like industrial monitoring, finance, and cloud systems. At any timestamp *t*, the observations form a column vector $x_{t}\in R^{N}$, where *N* denotes the number of monitored variables.

The training dataset—assumed to consist solely of normal behavior—is defined as follows:

$$X_{\mathrm{train}}=\left[x_{1},x_{2},\ldots ,x_{T_{\mathrm{train}}}\right]\in R^{N\times T_{\mathrm{train}}},$$

where $T_{\mathrm{train}}$ is the number of time steps in the training sequence.

Similarly, the test data is represented as follows:

$$X_{\mathrm{test}}=\left[x_{T_{\mathrm{train}}+1},\ldots ,x_{T_{\mathrm{train}}+T_{\mathrm{test}}}\right]\in R^{N\times T_{\mathrm{test}}}.$$

Given a historical window of length τ, we aim to learn a function $f:R^{N\times \tau }\to R$ that assigns a real-valued anomaly score s(t) at each timestamp *t*:

$$s\left(t\right)=f\left(x_{t-\tau +1},\ldots ,x_{t};\Theta \right),$$

where Θ denotes the learnable model parameters.

An anomaly is flagged if s(t) exceeds a threshold δ:

$$a\left(t\right)=\left\{\begin{array}{cc} 1 & \mathrm{if}\;s(t)>\delta ,\\ 0 & \mathrm{otherwise}, \end{array}\right.$$

where a(t)∈{0,1} indicates whether the system is anomalous at time *t*.

Training is performed exclusively on normal data. The learned model is expected to assign higher scores to test points that deviate from these normal patterns.

## 4. Methodology

### 4.1. Overview of the Framework

**Figure 2:**
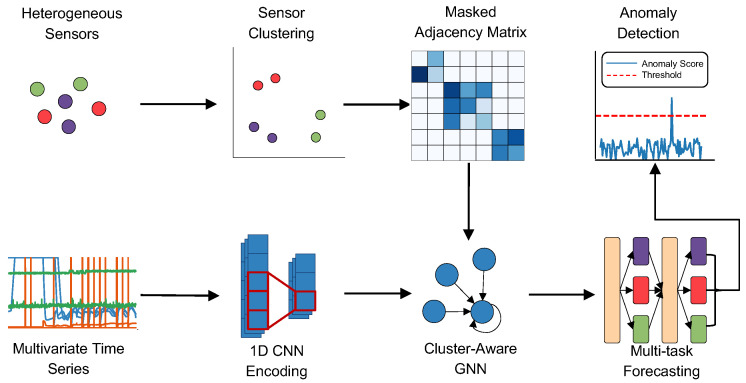
Overview of the MLAD framework. The pipeline begins with heterogeneous sensor inputs, which are then clustered into behaviorally similar sensor groups. Before training the GNN, a masked adjacency matrix is created to reflect connections within each sensor cluster. The input time series is encoded via 1D CNNs, then processed through a cluster-constrained GNN to model dependencies. Finally, a multi-task forecasting module predicts future values and anomalies are detected by evaluating the forecasting error through a PCA-based anomaly scoring mechanism.

To address the challenges of detecting anomalies in complex multivariate time-series data, we propose MLAD, a framework that integrates cluster-aware GNN and multi-task learning. The framework, shown in Figure 2, consists of four components: (1) sensor clustering, (2) GNN learning with cluster-constrained graph construction, (3) multi-task forecasting, and (4) a PCA-based anomaly detection module.

For a given multivariate time series, we begin by generating low-dimensional embeddings for each sensor’s time-series sequence using Uniform Manifold Approximation and Projection (UMAP) [26]. These embeddings capture the temporal characteristics of each sensor and are subsequently clustered using Density-Based Spatial Clustering of Applications with Noise (DBSCAN) [27]. The resulting clusters reveal groups of sensors with similar dynamic behavior and serve as structural priors for both graph construction and multi-task learning modules.

Building on the identified sensor clusters, we process the multivariate time series through a GNN module designed with cluster-aware graph construction. First, a 1D convolutional encoder extracts temporal features from each sensor’s sequence. These features are then passed to the GNN, where each sensor is represented as a node in a graph and edge weights between nodes are learned in a data-driven manner. To ensure robustness and meaningful connectivity, we apply a cluster-aware masking strategy that restricts edges to only form between nodes within the same cluster, reflecting the assumption that sensors in the same group exhibit stronger correlations.

To capture both common and cluster-specific temporal behaviors, we employ a multi-task forecasting head after the GNN module. This component consists of a set of shared layers that capture common temporal dynamics, along with task-specific layers tailored to each sensor cluster. By separating the forecasting tasks according to cluster membership, the model is able to learn both shared and specialized patterns across sensor clusters. The resulting predictions are then passed to a PCA-based anomaly detection module to compute anomaly scores.

The remainder of this section provides a detailed explanation of each component within the MLAD framework. Section 4.2 outlines the embedding and clustering process used to identify sensor groups. Section 4.3 describes the construction of the cluster-constrained graph and the graph neural network architecture. Section 4.4 details the multi-task forecasting module designed to handle both shared and cluster-specific patterns. Finally, Section 4.5 explains the PCA-based anomaly scoring mechanism used to identify deviations from expected behavior.

### 4.2. Sensor Clustering

To enhance structural awareness and improve the precision of anomaly detection, we begin with a clustering operation that identifies latent groupings of sensors based on their temporal dynamics. By uncovering these intrinsic relationships, the framework is better equipped to learn both general patterns shared across the system and specific dynamics unique to individual sensor clusters.

Given a multivariate training dataset $X_{\mathrm{train}}\in R^{N\times T_{\mathrm{train}}}$, where each row $x_{n}\in R^{T_{\mathrm{train}}}$ represents the time series for sensor *n*, we treat each $x_{n}$ as a high-dimensional sequence. Due to the long temporal sequences involved, we first project these time series into a lower-dimensional latent space to make them comparable. For this purpose, we employ UMAP [26], a non-linear dimensionality reduction technique that preserves both local and global data structure. The embedding of sensor *n* given by UMAP is

$$z_{n}=\mathrm{UMAP}\left(x_{n}\right)\in R^{d_{\mathrm{emb}}},\;\mathrm{for}\;n=1,\ldots ,N,$$

where $d_{\mathrm{emb}}\ll T_{\mathrm{train}}$ is the embedding dimension.

Specifically, UMAP builds a fuzzy topological graph in the original space by computing the edge probability between sensors *i* and *j* as follows:

$$p_{i|j}=\exp\left(-\frac{d\left(x_{i},x_{j}\right)-\rho_{i}}{\sigma_{i}}\right),$$

where $d(x_{i},x_{j})$ denotes the distance between the series, and $\rho_{i}$, $\sigma_{i}$ are local parameters controlling neighbor sensitivity. Based on this topological graph, the time series of sensors are then mapped into a low-dimensional embedding space, while preserving the relative distances among sensors. For further theoretical details, readers are referred to the original work [26].

Once embedded, we apply DBSCAN [27] on the set of embeddings ${\left\{z_{n}\right\}}_{n=1}^{N}$. DBSCAN clusters nearby points based on density connectivity and marks isolated points as noise. Formally, it assigns each sensor a discrete cluster label:

$$c_{n}=\mathrm{DBSCAN}\left(z_{n}\right),\;c_{n}\in \left\{1,2,\ldots ,C\right\},$$

where *C* is the total number of clusters discovered.

These cluster assignments serve as a structural prior for the remaining components of our model. They constrain the connectivity of the learned sensor graph (Section 4.3) and determine the organization of multi-task prediction heads (Section 4.4).

### 4.3. GNN Learning with Cluster-Constrained Graph Construction

With the sensor clusters identified in the previous stage, we proceed to construct a graph of sensors that leverages these structural priors by restricting connectivity to occur only within the same clusters. Specifically, we enforce that edges only form among sensors within the same clusters. This design enhances robustness by limiting information propagation to semantically related sensors.

As shown in Figure 2, multivariate time series is first passed through a one-dimensional convolutional encoder, inspired by the gated temporal convolutional structure proposed in [28]. The encoder is defined as

(1) $$Z_{\mathrm{enc}}=f_{C}\left(Z,\Phi_{C}\right)⊙f_{G}\left(Z,\Phi_{G}\right),$$

where $Z_{\mathrm{enc}}$ is the encoded features, Z is a linearly projected version of the raw input, $f_{C}$ and $f_{G}$ denote the convolution and gating functions, and ⊙ indicates element-wise multiplication.

Following the 1D convolutional encoding, the extracted representations are passed into a GNN module. To incorporate structural priors derived from sensor clustering, we construct a cluster-aware graph where each node corresponds to a sensor.

Specifically, we begin with initializing two sets of learnable node embeddings, $E_{1},E_{2}\in R^{N\times d}$. These embeddings are then projected independently via trainable matrices followed by non-linear activation:

(2) $${\tilde{E}}_{1}=\tanh\left(\alpha E_{1}W_{1}\right),\;{\tilde{E}}_{2}=\tanh\left(\alpha E_{2}W_{2}\right),$$

where $W_{1},W_{2}\in R^{d\times d}$ are learnable parameters, and α controls the activation sharpness.

To capture directional influence between sensors, we adopt an asymmetric similarity function inspired by [11] to compute a directed adjacency matrix $A\in R^{N\times N}$:

(3) $$A=\mathrm{ReLU}\left(\tanh\left(\alpha \left({\tilde{E}}_{1}{\tilde{E}}_{2}^{⊤}-{\tilde{E}}_{2}{\tilde{E}}_{1}^{⊤}\right)\right)\right),$$

This formulation yields a non-negative adjacency matrix where $A_{ij}$ indicates the directed influence from sensor *j* to sensor *i*.

Based on the raw adjacency matrix, a key innovation in our framework is the introduction of a masking mechanism to incorporate structural priors from the sensor clustering stage (Section 4.2). We hypothesize that functional correlations are more meaningful within clusters of similarly behaving sensors than across heterogeneous groups. To operationalize this, we define a binary mask $M\in {\left\{0,1\right\}}^{N\times N}$:

(4) $$M_{ij}=\left\{\begin{array}{cc} 1 & \mathrm{if}\;c_{i}=c_{j},\\ 0 & \mathrm{otherwise}, \end{array}\right.$$

where $c_{i}$ denotes the cluster assignment of sensor *i*. The masked graph is computed as

(5) $$A_{\mathrm{masked}}=A⊙M,$$

where ⊙ denotes element-wise multiplication.

This cluster-constrained design enforces connectivity among sensors in the same cluster and discourages spurious inter-cluster edges, promoting better alignment with real-world modular structures. Unlike prior methods that treat all sensor pairs uniformly, our cluster-aware masking introduces an explicit structural inductive bias.

To further reduce redundancy and improve efficiency, we sparsify the masked graph by retaining only the top-*k* strongest connections for each node. The resulting $A_{\mathrm{masked}}$ is a sparse, directed, and cluster-aware adjacency matrix.

With the masked adjacency matrix $A_{\mathrm{masked}}$ established, we proceed to perform directional message passing using a gated graph convolutional architecture. Let $Z_{\mathrm{enc}}$ denote the output of the CNN encoder, which serves as the input to the GNN.

Similar to [28], the GNN is composed of *K* propagation steps, and at each depth *k*, node representations are updated using a gated residual mechanism:

(6) $$H^{k+1}=\beta Z_{\mathrm{enc}}+\left(1-\beta \right){\tilde{A}}_{\mathrm{masked}}H^{k},\;k=0,1,\ldots ,K,$$

where ${\tilde{A}}_{\mathrm{masked}}$ is the row-normalized version of $A_{\mathrm{masked}}+I$ and β∈[0,1] is a learnable gating parameter.

After *K* iterations, the final output of the GNN is computed as a weighted sum across all propogation steps:

(7) $$H_{\mathrm{out}}=\sum_{k=0}^{K}H^{k}W^{k},$$

where each $W^{k}$ is a layer-specific learnable weight matrix.

To enhance the interaction between temporal and spatial features, our architecture stacks multiple layers of CNN encoders and cluster-constrained GNN modules. The CNN captures each sensor’s temporal patterns, while the GNN models dependencies among sensors. Residual connections are applied on each layer to facilitate more stable training by improving gradient flow. The final latent feature map is then passed to the multi-task learning module, which generates predictions used for anomaly detection.

### 4.4. Multi-Task Forecasting

To model both shared and cluster-specific patterns, we introduce a multi-task forecasting module—one of the central innovations of our framework. Unlike previous works [10,11] that apply a uniform predictor across all sensors, our approach decomposes forecasting into a set of parallel tasks—each corresponding to a distinct sensor cluster—thereby explicitly modeling cluster-wise heterogeneity.

The GNN output is first processed through a series of shared transformation layers designed to capture global features across all sensors. These shared features are then routed into cluster-specific branches, where each branch learns to specialize in the unique dynamics of its corresponding cluster of sensors. This design enables the model to combine generalization with localized specialization.

Formally, let Y be the input tensor to the forecasting module, and let c∈{1,…,C} represent the cluster assignments. We first compute a shared latent representation:

$$Y_{\mathrm{shared}}=\mathrm{ReLU}\left(W_{\mathrm{shared}}\cdot Y+b_{\mathrm{shared}}\right),$$

where $W_{\mathrm{shared}}$ and $b_{\mathrm{shared}}$ are learnable parameters. This operation projects the input into a shared latent space that encodes global forecasting cues.

Next, for each cluster c∈{1,…,C}, we apply a cluster-specific transformation to the relevant slice of the shared representation:

$$Y^{\left(c\right)}=\mathrm{ReLU}\left(W_{\mathrm{cluster}}^{\left(c\right)}\cdot Y_{\mathrm{shared}}^{\left(c\right)}+b_{\mathrm{cluster}}^{\left(c\right)}\right),$$

where $Y_{\mathrm{shared}}^{\left(c\right)}$ denotes the rows of $Y_{\mathrm{shared}}$ corresponding to sensors assigned to cluster *c*, and $\left(W_{\mathrm{cluster}}^{\left(c\right)},b_{\mathrm{cluster}}^{\left(c\right)}\right)$ are learnable parameters for that cluster. This structure allows each branch to adapt to the patterns specific to its sensor cluster.

To maintain consistency with the original sensor order, the outputs of the cluster-specific branches are reassembled using a function:

$$Y_{\mathrm{refined}}=\mathrm{Merge}\left({\left\{Y^{\left(c\right)}\right\}}_{c=1}^{C}\right),$$

which maps the cluster-wise predictions back to their respective sensor positions, producing a final forecasting vector aligned with the input.

When necessary, the forecasting head applies multiple layers of shared and cluster-specific transformations in succession, enabling a deeper interaction between global and localized representations. The final refined predictions $Y_{\mathrm{refined}}$ are forwarded to the anomaly detection module, where discrepancies between predicted and observed values are assessed to determine potential anomalies.

### 4.5. Anomaly Detection

Based on the forecasting results, we detect anomalies in the multivariate time series by analyzing PCA-reconstruction errors. Specifically, we compute the pointwise prediction error between the predicted and actual values, normalize these errors to account for variable-specific scale differences, and then apply PCA to reduce noise. An anomaly score is then derived by measuring the discrepancy between the original and PCA-reconstructed normalized error vectors.

Let $x^{t}$ and ${\hat{x}}^{t}$ denote the ground truth and predicted vectors at timestamp *t*, respectively. The absolute error is defined as

(8) $$e^{t}=\left|x^{t}-{\hat{x}}^{t}\right|,\;e^{t}\in R^{N\times 1}.$$

To account for variations in scale and variability across variables, each error is normalized using the median $\mu^{t}$ and interquartile range (IQR) $\sigma^{t}$ computed over a sliding window of length $W_{n}$:

(9) $${\tilde{e}}^{t}=\frac{e^{t}-\mu^{t}}{\sigma^{t}}.$$

Inspired by [11], we apply PCA to reduce noise in the normalized error signals. PCA is fitted on the set of validation errors ${\tilde{E}}_{v}\in R^{N\times T_{\mathrm{valid}}}$. From this, we compute the mean and covariance, followed by eigendecomposition:

$$\mathrm{cov}\left({\tilde{E}}_{v}\right)=U\Lambda U^{-1},$$

where U contains orthogonal eigenvectors and Λ is a diagonal matrix of eigenvalues.

For a test-time vector ${\tilde{e}}^{t}$, we subtract the validation mean and project it onto the PCA space defined by U. Retaining the top-*K* components, we reconstruct the denoised vector:

(10) $$P=\left({\tilde{e}}^{t}-\mathrm{mean}\left({\tilde{E}}_{v}\right)\right)U^{⊤},$$

(11) $$P_{K}=P\left[:,1:K\right],\;U_{K}=U\left[:,1:K\right],$$

(12) $${\tilde{e}}_{\mathrm{PCA}}^{t}=P_{K}U_{K}^{⊤}+\mathrm{mean}\left({\tilde{E}}_{v}\right).$$

The anomaly score s(t) is calculated as the $\ell_{1}$ distance between the original and reconstructed normalized error vectors:

(13) $$s\left(t\right)={∥{\tilde{e}}_{\mathrm{PCA}}^{t}-{\tilde{e}}^{t}∥}_{1}.$$

For thresholding, we classify a timestamp *t* as anomalous if its score exceeds the maximum anomaly score observed during the training period:

(14) $$s\left(t\right)>\max_{t\in T_{\mathrm{train}}}s\left(t\right).$$

This non-parametric approach removes the need for manual threshold tuning and is suitable for large-scale, heterogeneous time-series data. It assumes that training data reflects normal system behavior, enabling reliable anomaly detection through reconstruction residuals.

## 5. Experiments

In this section, we evaluate the effectiveness and generalization of our proposed MLAD framework for multivariate time-series anomaly detection. The experiments are designed to address the following research questions: (1) Can MLAD achieve superior anomaly detection performance across diverse and complex datasets? (2) How do the proposed cluster-constrained GNN learning and multi-task forecasting modules contribute to performance gains? To this end, we conduct comprehensive experiments on three public benchmark datasets, compare against strong baselines, and perform detailed ablation analyses.

### 5.1. Experimental Settings

**(1) Datasets:** We evaluate the effectiveness of our proposed MLAD framework on three widely-used benchmark datasets: SWaT, WaDi, and SMD.

- SWaT [8] is collected from a scaled-down version of a real-world industrial water treatment testbed. The dataset comprises 11 days of multivariate sensor readings, divided into 7 days for training (normal data only) and 4 days for testing. During testing, anomalies are labeled based on a series of simulated attack scenarios. To ensure consistency with previous studies [10,11], we follow a common preprocessing approach: the first 21,600 samples are removed, and the data is downsampled by taking the median value over 10 s intervals.
- WaDi [7] is an extended version of the SWaT dataset, representing a more complex and larger-scale water distribution system. The training set includes 14 days of normal operation, while the test set contains 2 days of labeled attack data. As with SWaT, we remove the first 21,600 samples and apply 10-s median downsampling.
- SMD [29] (Server Machine Dataset) consists of time-series readings from 28 servers, each with 38 variables. However, prior studies have shown that 16 of these machines exhibit significant concept drift, which can confound anomaly detection performance [30]. Following [11], we focus only on the 12 machines with stable distributions and report averaged results across these selected subsets.

**(2) Evaluation Metrics:** We assess the anomaly detection performance of MLAD and all baseline methods using two widely adopted metrics: Area Under the Receiver Operating Characteristic Curve (AUC-ROC) and Area Under the Precision–Recall Curve (AUC-PRC). AUC-ROC evaluates the model’s ability to distinguish between normal and anomalous samples across different thresholds by computing the area under the ROC curve, which plots the true positive rate (TPR) against the false positive rate (FPR). AUC-PRC focuses more on the positive (anomalous) class, which is particularly important in imbalanced datasets. It is computed as the Area Under the Precision–Recall Curve. Both metrics are threshold-independent and offer complementary insights into detection quality.

**(3) Baselines:** We compare MLAD with a comprehensive set of baselines categorized into three groups. (1) Classical statistical methods: PCA and KMeans provide simple, interpretable baselines for anomaly detection. (2) Deep reconstruction models: AutoEncoder and USAD [31] are unsupervised models that learn to reconstruct normal patterns, detecting anomalies via reconstruction error. (3) Graph and temporal models: MTAD-GAT [32] and GDN [10] incorporate graph neural networks and attention mechanisms to model complex inter-variable dependencies. THOC [14] applies hierarchical temporal clustering for one-class classification. CST-GL [11], a recent state-of-the-art method, constructs a correlation-aware spatial–temporal graph to model both feature and temporal structure. These baselines reflect the current landscape of multivariate time-series anomaly detection and provide rigorous points of comparison.

**(4) Implementation Details:** We implemented MLAD using PyTorch 2.6.0 and conducted all experiments on a single NVIDIA RTX 3090 GPU (NVIDIA Corporation, Santa Clara, CA, USA). Model hyperparameters were selected based on validation performance and alignment with prior studies. For sensor clustering, we set the UMAP embedding dimension to 5, and applied DBSCAN with an epsilon of 2 and a minimum samples threshold of 10 to identify sensor clusters. The temporal encoder consists of two convolutional layers, and the GNN module uses a 256-dimensional node embedding and two propagation layers to model interactions among sensors in the same clusters. The multi-task forecasting head includes two shared layers for global dynamics and two sets of cluster-specific layers, each with one fully connected layer per cluster. We optimized the model using the Adam optimizer with a learning rate of 0.0005 and a batch size of 64, and trained using a masked mean squared error loss that selectively penalizes errors on observed values. Following prior work [29,30], we set the validation ratio to 0.1 for SWaT and WADI, and 0.3 for SMD. The sliding window sizes were set to w=5 for SWaT and WADI, and w=100 for SMD, consistent with previous studies [10,11]. The full model training for each dataset was completed within a few hours, while the computational cost for the UMAP and DBSCAN clustering was minimal, typically finishing within minutes. Overall, the approach remains computationally efficient and suitable for practical, moderate-scale deployments.

### 5.2. Overall Anomaly Detection Results

As shown in Table 1, MLAD achieves strong performance across all three benchmark datasets in both AUC-ROC and AUC-PRC, consistently outperforming state-of-the-art baselines. These results validate the effectiveness of our cluster-aware GNN and multi-task forecasting design in capturing both shared and cluster-specific temporal dynamics. In particular, MLAD’s improvements are most pronounced in AUC-ROC.

On the SWaT dataset, MLAD achieves an AUC-ROC of 0.8583 and an AUC-PRC of 0.7570. This slightly surpasses the previous top performer CST-GL (0.8520/0.7628) in AUC-ROC while remaining highly competitive in AUC-PRC. Compared to THOC, the second-best in AUC-ROC (0.8380) and AUC-PRC (0.7440), MLAD delivers notable gains. Even against MTAD-GAT—the third-best in AUC-PRC (0.7176)—our model demonstrates a consistent performance margin.

On the more complex WaDi dataset, MLAD achieves the highest AUC-ROC (0.8327), slightly edging out CST-GL (0.8283). While its AUC-PRC (0.5267) falls just below CST-GL (0.5477), it still ranks among the top performers and shows a significant margin over third-place methods such as PCA and USAD.

For SMD, MLAD again ranks first with an AUC-ROC of 0.8703 and AUC-PRC of 0.5204. It edges out CST-GL (0.8604/0.5132) and MTAD-GAT (0.8576/0.5057), reaffirming its ability to generalize across heterogeneous sensor environments.

Overall, MLAD delivers consistently strong performance across varied domains, confirming its robustness and adaptability to both structured industrial systems and high-dimensional cloud environments.

### 5.3. Ablation Study

**Figure 3:**
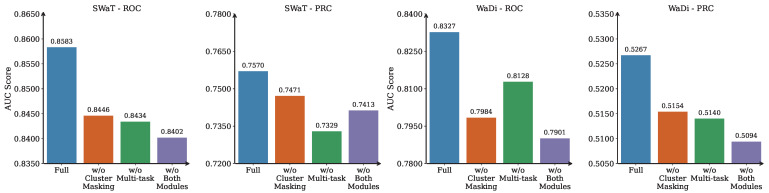
Ablation study results on SWaT and WaDi datasets. Bars represent average AUC-ROC and AUC-PRC scores across 3 runs. Removing either the cluster masking or multi-tasking module leads to a performance drop, validating their contribution to the full MLAD framework.

To assess the individual contributions of MLAD’s key architectural components, we conduct an ablation study by systematically removing the cluster masking mechanism, the multi-task forecasting module, and both simultaneously. The results are reported in Figure 3, which shows average AUC-ROC and AUC-PRC scores on SWaT and WADI across five independent runs. This evaluation allows us to quantify the importance of each module in isolation and to better understand how they interact to support robust anomaly detection under complex multivariate dynamics.

**(1) Contribution of Cluster Masking.** The cluster masking mechanism is a core structural prior in MLAD, designed to restrict graph connectivity within coherent clusters of sensors. This masking encourages message passing only among sensors that share similar temporal dynamics, as determined through UMAP and DBSCAN clustering. By incorporating this inductive bias, the model avoids propagating signals between loosely correlated or unrelated variables. As shown in our experiments, removing this module leads to performance degradation on both datasets—dropping from 0.8583 to 0.8446 AUC-ROC and from 0.7570 to 0.7471 AUC-PRC on SWaT. This indicates that while the GNN can still learn inter-sensor relationships in a data-driven way, the lack of structured guidance weakens its precision, especially for rare anomalies.

The impact is even more substantial on the WaDi dataset, where structural complexity and sensor heterogeneity are higher. Here, the model without cluster masking achieves only 0.7984 AUC-ROC and 0.5154 AUC-PRC, down from 0.8327 and 0.5267 respectively. This supports the intuition that enforcing connectivity within clusters is especially important in large-scale, multi-module systems. Without this masking, the GNN risks overfitting to noisy or misleading correlations across sensor subsystems.

**(2) Effectiveness of Multi-task Forecasting Module.** Our multi-task forecasting module aims to model both global temporal dynamics and cluster-specific behavior patterns. The shared layers capture system-wide trends, while the cluster-specific branches allow for specialization based on sensor clustering. Ablating this module forces the model to apply the same forecasting logic to all sensors, regardless of their group patterns. On SWaT, this simplification results in performance dropping to 0.8434 AUC-ROC and 0.7329 AUC-PRC. Compared to the full model, this highlights a diminished capacity to detect anomalies that emerge in highly localized or cluster-specific ways.

WaDi results reflect a similar trend. The AUC-ROC drops to 0.8128 and AUC-PRC to 0.5140. Although this drop is slightly smaller than that caused by removing the cluster masking, it still illustrates the importance of the multi-task design. In environments where sensor modules operate under different physical or logical regimes, applying a single forecasting head fails to capture the nuance of localized failures.

**(3) Combined Effect Analysis.** To evaluate the complementary role of the two modules, we analyze the performance when both cluster masking and multi-task forecasting are removed. In this ablated variant, the model relies solely on unconstrained graph learning and a single forecasting head. Performance degrades to 0.8402 AUC-ROC and 0.7413 AUC-PRC on SWaT, and to 0.7901 AUC-ROC and 0.5094 AUC-PRC on WaDi. The combined removal results in the worst scores across all configurations, emphasizing that each module plays a non-redundant role in the MLAD pipeline.

This outcome aligns with the framework’s conceptual foundation: cluster masking shapes the graph’s structure by embedding sensor group information, while multi-task forecasting the separation between sensor clusters during prediction. When both constraints are lifted, the model’s inductive bias weakens considerably, leading to poorer generalization and noisier anomaly scores. Importantly, this supports our design philosophy of modular integration—each component addresses a distinct modeling challenge, and their synergy results in more robust detection under complex real-world conditions.

**(4) Summary of Findings.** The ablation study provides clear and consistent evidence supporting the architectural choices made in MLAD. Both the cluster masking strategy and the multi-task forecasting module contribute significantly to overall performance. Their removal leads to noticeable degradation in both AUC-ROC and AUC-PRC. Together, these experiments demonstrate that MLAD’s modular design is not only interpretable but also empirically effective. The cluster masking module injects structural priors that improve GNN message passing, while the multi-task forecasting head enables flexible adaptation to heterogeneous sensor dynamics.

## 6. Conclusions, Limitation, and Future Direction

In this paper, we introduced MLAD, a novel framework for multivariate time-series anomaly detection that integrates cluster-constrained graph neural network with multi-task forecasting. MLAD leverages unsupervised clustering to identify behaviorally coherent sensor groups. These clusters serve as structural priors for two key components: a cluster-constrained GNN that models sensor relationships within each cluster, and a multi-task forecasting module that captures both global system dynamics and cluster-specific patterns.

Through extensive experiments on three benchmark datasets—SWaT, WaDi, and SMD—MLAD consistently outperforms strong baseline methods. Our framework not only demonstrates superior detection accuracy but also shows robustness across heterogeneous environments with varying system complexity. Ablation studies further validate the complementary contributions of the clustering-based GNN learning and the multi-task forecasting design.

Despite its strong empirical performance, MLAD has several limitations. First, the sensor clustering step is performed offline and remains fixed during model training. This may limit adaptability in dynamic environments where sensor relationships evolve over time. Second, although the anomaly scoring mechanism is effective, it currently treats all prediction errors equally, without explicitly incorporating sensor cluster information. Addressing these limitations could further enhance the flexibility, precision, and interpretability of the framework in real-world deployments. Moreover, our current pipeline performs clustering and anomaly detection in sequence, which limits its suitability for real-time or continuously evolving environments.

Moving forward, several promising research directions emerge. One is to integrate clustering into the training pipeline, enabling the model to jointly learn sensor clustering and anomaly detection in an end-to-end manner. Another is to enhance the interpretability of the framework by introducing mechanisms that explain why anomalies are detected, possibly at the level of sensor clusters or graph structure. Lastly, incorporating cluster-specific anomaly scoring may further improve the model’s sensitivity and robustness in complex, real-world deployments.

In summary, MLAD represents an effective and robust approach to anomaly detection in multivariate time series, particularly in systems characterized by heterogeneous sensor behavior. By bridging sensor clustering with multi-task learning techniques, it offers strong foundation for future innovation.

## Figures and Tables

**Table 1:** Average AUC performance (±standard deviation) of five experimental runs on three benchmark datasets. The best, second best, and third best results in each column are marked in **bold**, with * and † respectively.

	SWaT		WADI		SMD	
	**AUC-ROC**	**AUC-PRC**	**AUC-ROC**	**AUC-PRC**	**AUC-ROC**	**AUC-PRC**
PCA	0.8257 ± 0.0000	0.7298 ± 0.0000	0.5597 ± 0.0000	† 0.2731 ± 0.0000	0.6742 ± 0.0000	0.2189 ± 0.0000
Kmeans	0.7391 ± 0.0000	0.2418 ± 0.0000	† 0.6030 ± 0.0000	0.1158 ± 0.0000	0.5855 ± 0.0000	0.1308 ± 0.0000
AutoEncoder	0.8311 ± 0.0088	0.7224 ± 0.0094	0.5291 ± 0.0285	0.2210 ± 0.0205	0.8270 ± 0.0008	0.4388 ± 0.0046
USAD	0.8213 ± 0.0056	0.7087 ± 0.0055	0.5535 ± 0.0103	0.1945 ± 0.0008	0.7888 ± 0.0077	0.4686 ± 0.0011
MTAD-GAT	0.8261 ± 0.0040	0.7176 ± 0.0043	0.4119 ± 0.0295	0.0729 ± 0.0013	† 0.8576 ± 0.0035	† 0.5057 ± 0.0082
THOC	† 0.8380 ± 0.0051	† 0.7440 ± 0.0063	0.4840 ± 0.0112	0.1440 ± 0.0020	0.8512 ± 0.0045	0.4852 ± 0.0063
GDN	0.8124 ± 0.0177	0.7135 ± 0.0035	0.4725 ± 0.0056	0.0521 ± 0.0070	0.8443 ± 0.0150	0.4684 ± 0.0142
CST-GL	* 0.8520 ± 0.0022	**0.7628** ± 0.0032	* 0.8283 ± 0.0179	**0.5477** ± 0.0197	* 0.8604 ± 0.0131	* 0.5132 ± 0.0273
**MLAD (ours)**	**0.8583** ± 0.0025	* 0.7570 ± 0.0050	**0.8327** ± 0.0040	* 0.5267 ± 0.0034	**0.8703** ± 0.0078	**0.5204** ± 0.0031

## Data Availability

The datasets used in this study are publicly available. SWaT and WaDi can be accessed via iTrust Singapore, and SMD is available at: https://github.com/NetManAIOps/OmniAnomaly (accessed on 1 February 2025).
